# Expression profiling dataset of competing endogenous RNA in pre-eclampsia

**DOI:** 10.1016/j.dib.2019.104795

**Published:** 2019-11-12

**Authors:** Xiaopeng Hu, Xinyue Li, Geng G. Tian, Huijuan Zhang, Weiwei Cheng, Ji Wu

**Affiliations:** a Bio-X Institutes, Shanghai Jiao Tong University, Shanghai, 200240, China; b International Peace Maternity & Child Health Hospital, Shanghai Jiao Tong University School of Medicine, Shanghai, 200030, China; c Key Laboratory of Fertility Preservation and Maintenance of Ministry of Education, Ningxia Medical University, Yinchuan, 750004, China; d Shanghai Key Laboratory of Reproductive Medicine, Shanghai, 200025, China

**Keywords:** Competing endogenous RNA, Pre-eclampsia, Expression profiling dataset, circRNA, lncRNA, mRNA

## Abstract

This data descriptor is an extended version of the methodologies which have been described in a related paper [1]; its purpose is to disseminate the raw data and analyzed data produced in this experiment. For more insight please see the research article, “competing endogenous RNA expression profiling in pre-eclampsia identifies hsa_circ_0036877 as a potential novel blood biomarker for early pre-eclampsia” (Xiaopeng Hu and Junping Ao, 2018) [1]. Using microarray analysis, we investigated competing endogenous RNA (ceRNA) expression profiles in placentas of women with severe pre-eclampsia (SPE) and normal pregnancies. Competing endogenous RNA (ceRNA) refer to RNA transcripts, such as messenger RNA (mRNA), long noncoding RNA (lncRNA), and circular RNA (circRNA) that can regulate each other by competing for the same pool of miRNAs. CircRNAs, lncRNAs, and mRNAs differentially expressed between human normal and SPE placentas were obtained in the study. Metadata files were submitted to the Gene Expression Omnibus repository (GEO), with GEO accession number GSE102897. These data are potential useful for further study on the pathogenesis of PE and early prediction of PE onset.

**Table:** Specifications Table

Subject area	Medicine
More specific subject area	Pre-eclampsia (PE)
Type of data	Table and figure
How data was acquired	Microarray
Data format	Raw and analyzed data
Parameters for data collection	The human placentas of controls and SPE were randomly devided into 3 groups respectively.
Description of data collection	Total RNA were extracted from the placental tissue for analysis of their circRNA, lncRNA, and mRNA profiles using Agilent Human (4 × 180K) GeneChip circRNA and lncRNA arrays.
Data source location	Shanghai, china
Data accessibility	Data was submitted to the Gene Expression Omnibus repository (GEO) (https://www.ncbi.nlm.nih.gov/geo/query/acc.cgi?acc=GSE102897), with GEO accession number GSE102897.
Related research article	Hu X, Ao J, Li X, Zhang H, Wu J, Cheng W. Competing endogenous RNA expression profiling in pre-eclampsia identifies hsa_circ_0036877 as a potential novel blood biomarker for early pre-eclampsia. Clinical epigenetics 2018, **10:** 48.

**Table:** 

**Value of the Data** • The circRNA, lncRNA and mRNA expression profiling were performed simultaneously, which is generally believed to be one of the major advantages of this research. It is believed that this data is useful for more precise investigation of the interaction between ceRNAs in the process of PE onset. • It could also help PE researchers to resolve the issue of how ceRNAs are involved in the pathogenesis of PE. • The data could be used to analyse predicted targets associated to treatment of PE as it concerns maternal and child health, as well as to identify ceRNAs as early clinical biomarkers of PE. • After bioinformatics analysis, this dataset shows all differentially expressed ceRNAs between human normal and SPE placentas in tables. This dataset also shows all items of functional enrichment and prediction of ceRNAs interaction in tables.

## 1 Data

**Fig. 1:**
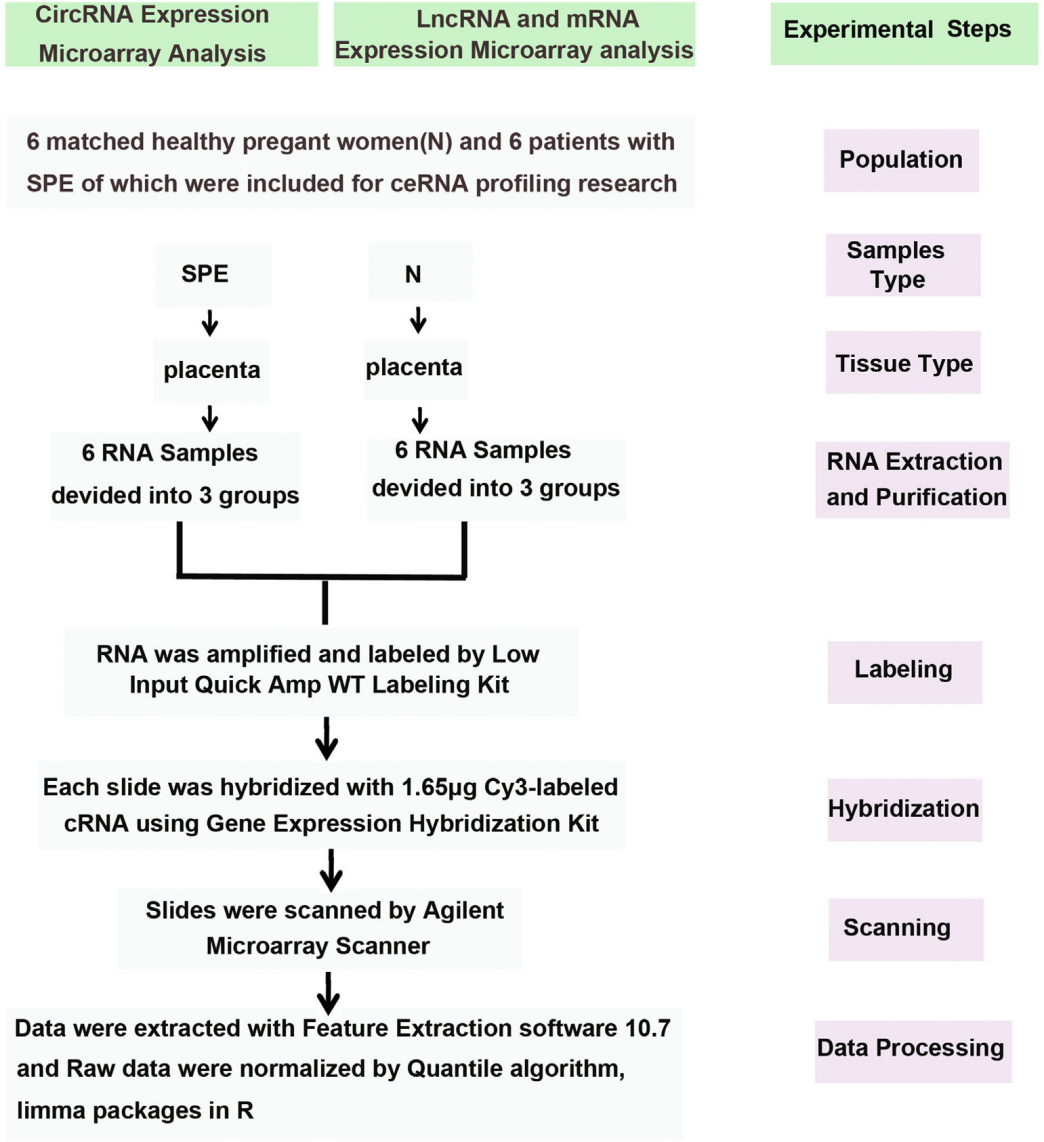
A Flow chart diagram illustrating the steps in circRNA, lncRNA and mRNA expression profiling analysis.

**Fig. 2:**
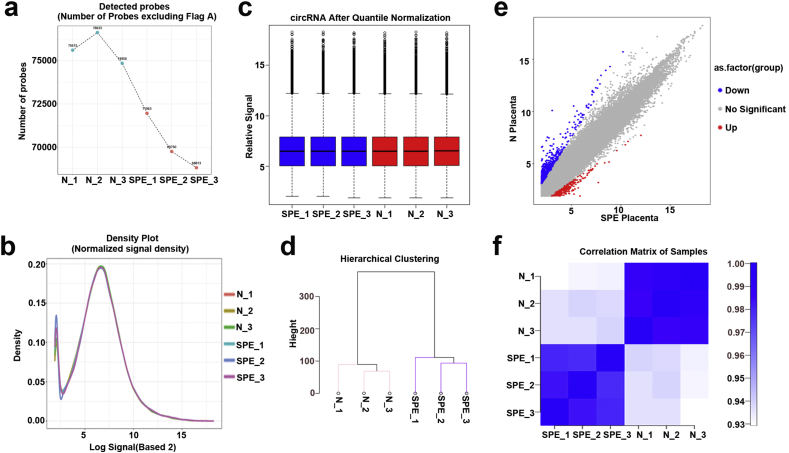
**Quality check of circRNA profiling data.** (a) The total number of circRNAs probes which were detected in each group. (b) The circRNA expression density for each group was analyzed. (c) The distribution of circRNA microarray data for each group was analyzed by Box plot (d) Hierarchical clustering analysis between groups. (e)Scatter plot showed the significantly differential expression of circRNAs compared SPE with normal placenta. (f) Generating correlation matrix based on the comparison of circRNA expression pattern between groups to show a high degree of repeatability between groups.

**Fig. 3:**
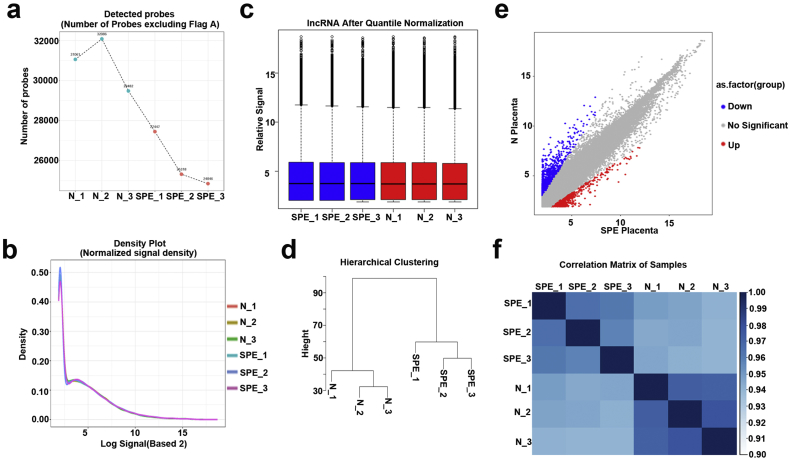
**Quality check of lncRNA profiling data.** (a) The total number of lncRNAs probes which were detected in each group. (b) The lncRNA expression density for each group was analyzed. (c) The distribution of lncRNA profiling data for each group was analyzed by Box plot (d) Hierarchical clustering analysis between groups. (e) Scatter plot showed the significantly differential expression of lncRNAs compared SPE with normal placenta. (f) Generating correlation matrix based on the comparison of lncRNA expression pattern between groups to show a high degree of repeatability between groups.

**Fig. 4:**
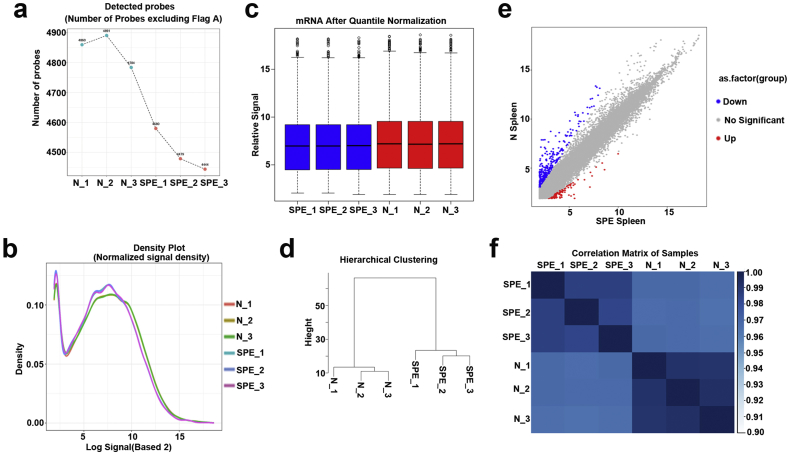
**Quality check of mRNA profiling data.** (a) The total number of gene probes which were detected in each group. (b) The gene expression density for each group was analyzed. (c) The distribution of mRNA profiling data for each group was analyzed by Box plot (d) Hierarchical clustering analysis between groups. (e) Scatter plot showed the significantly differential expression of genes compared SPE with normal placenta. (f) Generating correlation matrix based on the comparison of gene expression pattern between groups to show a high degree of repeatability between groups.

**Fig. 5:**
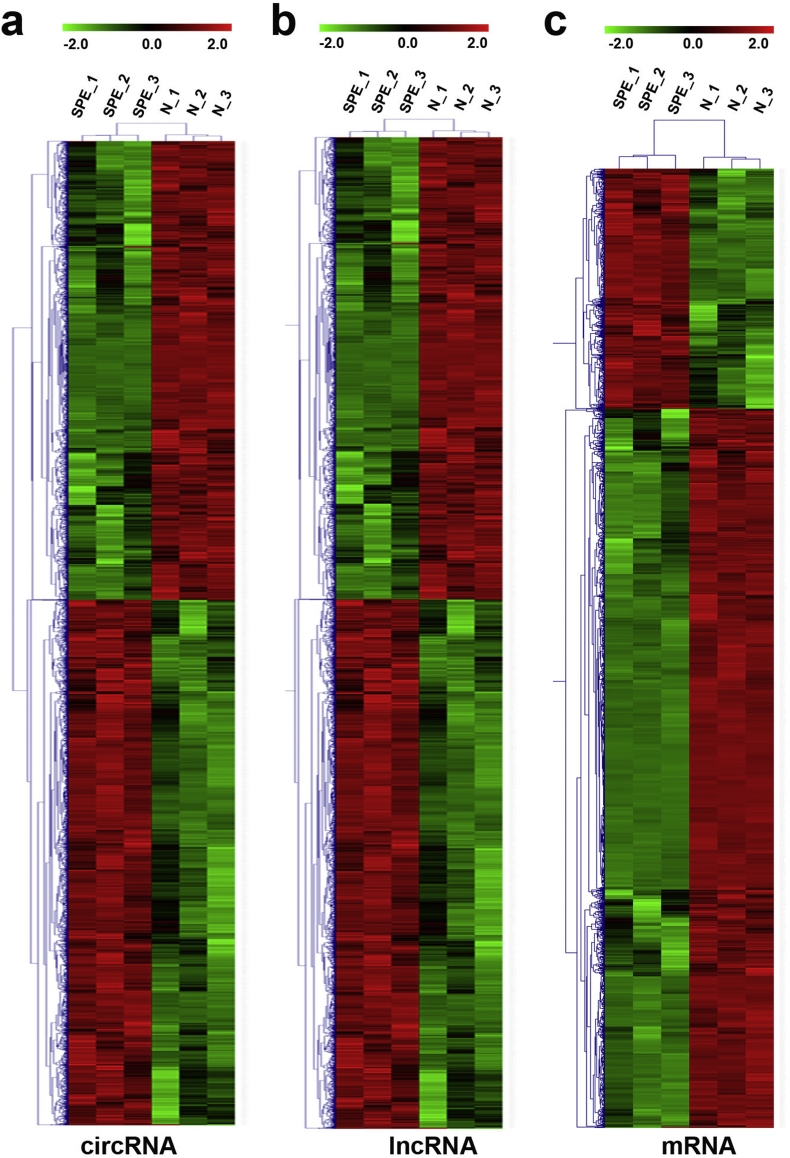
**Heat map shows hierarchical clustering of dysregulated ceRNAs between SPE and normal placentas.** (a) Hierarchical clustering shows the dysregulated circRNAs between SPE and normal placentas. (b) Hierarchical clustering shows the dysregulated lncRNAs between SPE and normal placentas. (c) Hierarchical clustering shows the dysregulated genes between SPE and normal placentas.

The dataset contains original and analyzed data obtained through the circRNA, lncRNA, mRNA expression microarray analysis of 12 placental samples, which were collected from six patients with SPE and six healthy pregnant women (Fig. 1). The original and normalized data of all SPE/normal placental samples have been deposited in the NCBI GEO repository with accession number GSE102897 for the circRNA, lncRNA and mRNA expression microarrays. The metadata record associated with the samples is shown in Table 1 and demographic characteristics of Participants were shown in related work [1]. Analyzed data is shown in tables (Tables S1–12). Based on the experimental procedure, a total new set of figures (Fig. 2, Fig. 3, Fig. 4, Fig. 5) were generated and quality controls were used to generate the dataset. The ceRNA dataset has little inter-sample variation in the expression of circRNAs, lncRNAs, and mRNAs among the placentas of pregnant women with PE or normal. The findings also suggest that the levels of inter-PE/normal sample variation and/or technical variation were low.

**Table 1:** Sample description for ceRNAs expression array.

Sample name	Source	Organism	Tissue	Age	Protocol 1	Protocol 2	Data
SPE_1	Severe Preeclampsia	Homo sapiens	Placenta	25-35y	RNA extraction	circRNA array IncRNA and mRNA array	GSM2747897
SPE_2	Severe Preeclampsia	Homo sapiens	Placenta	25-35y	RNA extraction	circRNA array IncRNA and mRNA array	GSM2747898
SPE_3	Severe Preeclampsia	Homo sapiens	Placenta	25-35y	RNA extraction	circRNA array IncRNA and mRNA array	GSM2747899
N_1	Normal human	Homo sapiens	Placenta	25-35y	RNA extraction	circRNA array IncRNA and mRNA array	GSM2747900
N_2	Normal human	Homo sapiens	Placenta	25-35y	RNA extraction	circRNA array IncRNA and mRNA array	GSM2747901
N_3	Normal human	Homo sapiens	Placenta	25-35y	RNA extraction	circRNA array IncRNA and mRNA array	GSM2747902

## 2 Experimental design, materials, and methods

These methods are expanded versions of descriptions in our related work [1].

### 2.1 Experimental design

As shown in Fig. 1, in this study, we included six patients with SPE and six healthy pregnant women as controls. The placentas of controls and SPE were randomly devided into 3 groups respectively. Total RNA were extracted from the placental tissue for analysis of their circRNA, lncRNA, and mRNA profiles using Agilent Human (4 × 180K) GeneChip circRNA and lncRNA arrays. The GeneChip circRNA array was designed to interrogate all sequences of circRNA in Circbase (88,371) [2], which is the updated international mainstream database of human circRNAs. The lncRNA array used lncRNA and mRNA probes derived from the updated version of a reference genome (human/GRCh38; hg38) [3], covering the following databases of lncRNA: GENCODE V21, Ensembl, UCSC, NONCODE, LNCipedia, and lncRNAdb. All lncRNA and mRNA sequences from the databases were aligned by Biological Information Experts, and then the redundant sequences were eliminated. The precise lncRNA (68,423) and mRNA (18,853) sequences were applied for specific probe design.

### 2.2 Samples and setting

The Ethics Committee of the International Peace Maternity and Child Health Hospital (Shanghai Jiaotong University, Shanghai, China) approved this research and prior written informed consent was signed by all participants to participate. From December 2015 to November 2017, Placental samples were taken from 6 Normal and 6 SPE Chinese pregnant women at the above-mentioned hospital, whose the demographic characteristics were shown in related work [1]. Each placental sample was stored at −80 °C until use and taken from the same representative region of the central portion of tissue at the lower third of the placenta, near the maternal side, which mainly contained cytotrophoblasts, syncytiotrophoblasts, and villous interstitium. In this research, patients including those with no history of hypertension, diabetes, or kidney disease were evaluated by an experienced obstetrician. SPE is defined as a higher blood pressure >160 mmHg systolic or >110 mmHg diastolic with protein urea of ≥300–5000 mg/24h, in accordance with the guidelines of the American College of Obstetricians and Gynecologists (ACOG) [4].

### 2.3 RNA extraction from placental samples

Using TAKARA RNAiso Plus#9109, total RNA was extracted following to the manufacturer's protocol.

### 2.4 RNA amplification and labeling

For RNA amplification and labeling, we used a Low Input Quick Amp WT Labeling Kit (Cat# 5190-2943; Agilent Technologies) according to the manufacturer's protocol and then purified by RNeasy Mini kit (Cat# 74106; QIAGEN, GmBH, Germany).

### 2.5 Quality control of the microarray

The coefficient of variation (CV) was applied as quality control and validation method for the analysis of microarray assays.

### 2.6 Microarray analysis

Each slide was hybridized with 1.65 μg of Cy3-labeled cRNA using a Gene Expression Hybridization Kit (Cat# 5188-5242; Agilent Technologies) in a Hybridization Oven (Cat# G2545A; Agilent Technologies) at 55 °C, 20 rpm for 17 hours according to the manufacturer's instructions. After hybridization, slides were washed in stain dishes (Cat# 121; Thermo Shandon, Waltham, MA, USA) with a Gene Expression Wash Buffer Kit (Cat# 5188–5327; Agilent Technologies).

Slides were scanned by an Agilent Microarray Scanner (Cat# G2565CA; Agilent Technologies) with the default settings (Agilent Technologies). Microarray data were feature extracted by Feature Extraction software 10.7 (Agilent Technologies). Raw data were normalized by the quantile algorithm of limma package in R.

To visualize the whole set of significant ceRNAs expression in PE compared to normal participants, hierarchical clustering in MeV_4_9_0 was used. The p-value was calculated by a right-sided hypergeometric test. Benjamini–Hochberg adjustment was used to correct for multiple tests. An adjusted p-value of <0.05 indicated a statistically significant deviation from the expected distribution. Gene-enrichment and functional annotation analyses for the significant probe list were performed using DAVID (http://david.abcc.ncifcrf.gov/home.jsp) [5,6].

## 3 Technical validation

### 3.1 Checked the RNA quality of samples

Using a Bioanalyzer 2100 (Agilent, CA, USA), RNA Integrity Number (RIN) was determined to evaluate RNA integrity. All RIN values of placental samples are greater than 7.0 and the ratio of 28S RNA to18S RNA is greater than 1.9.

### 3.2 Quality check of ceRNA profiling data

For this study, we confirmed the quality of the circRNA profiling data. The following methods were used: the number of circRNAs probes detected (Fig. 2a), the expression density (Fig. 2b)and the distribution of the normalized signal values (max, min, 1st Qu, 3rd Qu, Median) (Fig. 2c) for each group were analyzed; Correlation and hierarchical clustering analysis between groups were performed (Fig. 2d–f). In the same way, we confirmed the quality of the lncRNA and mRNA expression microarray analysis by analysing the number of detected probes (Fig. 3, Fig. 4a), the expression density (Fig. 3, Fig. 4b) and the distribution of the normalized signal values (max, min, 1st Qu, 3rd Qu, Median) (Fig. 3, Fig. 4c) for each group. Accordingly, Correlation and hierarchical clustering analysis between groups were also performed (Fig. 3, Fig. 4d–f).

### 3.3 Validation of mRNA, lncRNA and circRNA expression

As shown, circRNAs (Fig. 5a, Table S1), lncRNAs (Fig. 5b, Table S2) and genes (Fig. 5c, Table S3) showed significant changes in transcript expression between SPE and Normal placentas.

### 3.4 Functional enrichment analysis and prediction of ceRNA interaction

By DAVID analysis, Gene-enrichment, functional annotation and Pathway -enrichment were shown in Tables S4–9. By using cytoscape, ceRNA interaction between circRNA, lncRNA and mRNA were predicted, which were shown in Tables S10–12.
